# Supplementary material for: Cytoplasmic Kinase Network Mediates Defense Response to Spodoptera litura in Arabidopsis
Source: Plants (Basel). 2023 Apr 24;12(9):1747. doi: 10.3390/plants12091747 (PMC10180863; doi:10.3390/plants12091747)
Supplement: Supplementary file 1 [file plants-12-01747-s001.zip › plants-2294875-supplementary.pdf]

Table S1. Primers.

Purpose	Gene/clone/plasmid	Gene ID	Forward primer sequence (5′ to 3′)	Reverse primer sequence (5′ to 3′)	Rmarks
Gene cloning	*PBL27*	At5g18610	CACCATGAGTGGGTGTTTGCCTTGT	GTCATTTGTACTATCAAAGCTGC	clone into pENTR/D-TOPO
	CRK2	At3g19100	<u>CCACCCACCACCACCA</u>ATGGGAGGCT	<u>TCCAGCACTAGCTCCAGA</u>TCGCGTTTT	without stop codon, Underline: S1 or T1-linker sequence
	CRK3	At2g46700	<u>CCACCCACCACCACCA</u>ATGGGGCAAT	<u>TCCAGCACTAGCTCCAGA</u>GCGAGGTC	without stop codon, Underline: S1 or T1-linker sequence
	CPK3	At4g23650	<u>CCACCCACCACCACCA</u>ATGGGCCACAGACACAGCAA	<u>TCCAGCACTAGCTCCAGA</u>CATTCTGCGTCGGTTTGGCA	without stop codon, Underline: S1 or T1-linker sequence
	CPK13	At3g51850	<u>CCACCCACCACCACCA</u>ATGGGAAACTGTTGCAGATC	<u>TCCAGCACTAGCTCCAGA</u>TTCGTTGCCTAGGTTCAAAG	without stop codon, Underline: S1 or T1-linker sequence
	ERF13	At2g44840	<u>CCACCCACCACCACCA</u>ATGAGCTCATCTGATTCCGT	<u>TCCAGCACTAGCTCCAGA</u>TTATATCCGATTATCAGAAT	include stop codon, Underline: S1 or T1-linker sequence
	pDONRzeo_cloning_2nd PCR		GGGGACAAGTTTGTACAAAAAAGCAGGCTT<u>CCACCCACCACCA</u>ATG	GGGGACCACTTTGTACAAGAAAGCTGGGTC<u>TCCAGCACTAGCTCCAGA</u>	Underline: S1 or T1-linker sequence, clone into pDONRzeo
Promoter region cloning	*PDF1.2*	At5g44420	TTATAATGTTCGGTTTTTCC	TATTTCTTCACACAACAC	
RT-qPCR	*PDF1.2*	At5g44420	TGTTCTCTTTGCTGCTTTCG	GTTGCATGATCCATGTTTGG	
	ERF13	At2g44840	GGATGTACTTCCGTAACCCGA	GGCATCACGGAGAGTGTTGTA	
	ACT8	At1g49240	TGCTGGTCGTGACCTTACTG	CGAGGTCTCCATCTCTTGCT	

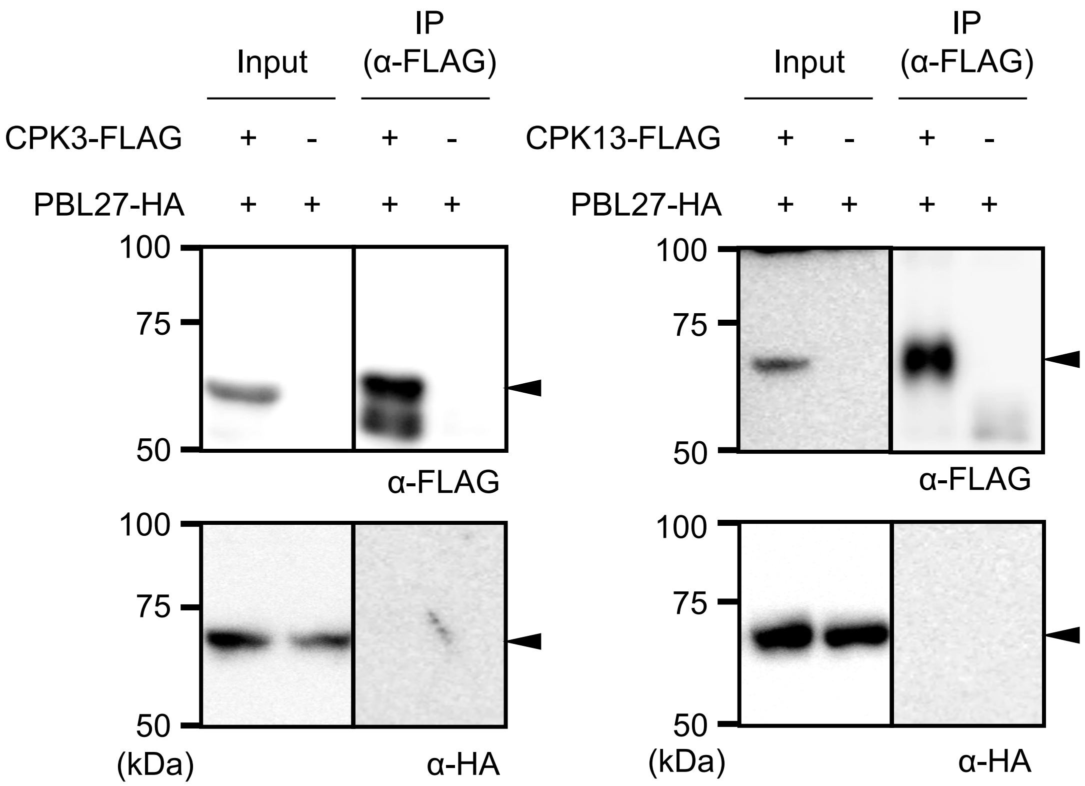
IP
Input
(α-FLAG)
CPK3-FLAG + - + -
PBL27-HA + + + +
100
75
50
α-FLAG
100
75
50
(kDa)
α-HA
IP
Input
(α-FLAG)
CPK13-FLAG + - + -
PBL27-HA + + + +
100
75
50
α-FLAG
100
75
50
(kDa)
α-HA

Fig. S1

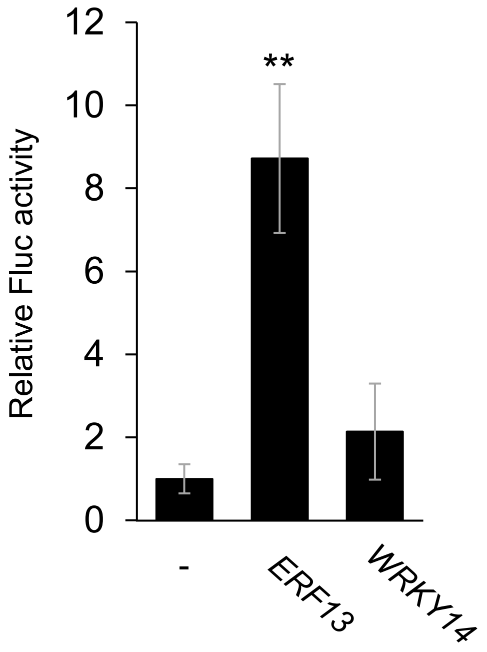

Fig. S2

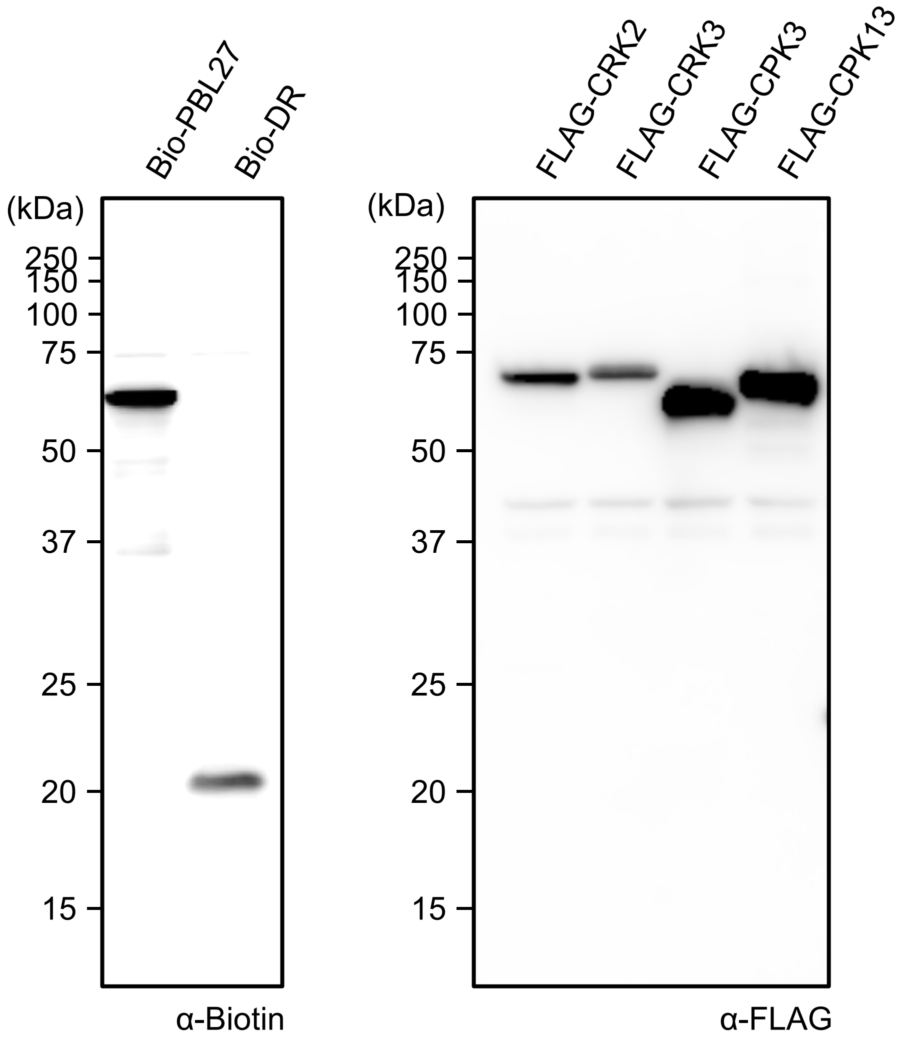
Bio-PBL27
Bio-DR
(kDa)
250
150
100
75
50
37
25
20
15
α-Biotin
FLAG-CRK2
FLAG-CRK3
FLAG-CPK3
FLAG-CPK13
(kDa)
250
150
100
75
50
37
25
20
15
α-FLAG

Fig. S3

Figure S1. Interactions of PBL27 and CPKs. A pair of FLAG-tagged CPK3 or CPK13 (CPK3-FLAG or CPK13-FLAG) and HA-tagged PBL27 (PBL27-HA) were expressed in *Nicotiana benthamiana* leaf cells. Total proteins extracted from the leaves were immunoprecipitated using anti-FLAG-tag magnetic beads, subjected to SDS-PAGE, and probed with anti-HA or anti-FLAG antibody as a primary antibody. Arrowheads indicate the predicted target signals.

Figure S2. Transient activation of a firefly luciferase reporter gene under the control of *PDF1.2* promoter according to coexpression with or without (-) *ERF13* or *WRKY14* in arabidopsis wild-type leaf protoplasts. Data represent the mean and standard error (n = 4). Data marked with asterisks are significantly different from those obtained without *ERF13* or *WRKY14* (-), based on an ANOVA with Holm's sequential Bonferroni post-hoc test (**, $P < 0.01$).

Figure S3. Recombinant proteins used for AlphaScreen. Immunoblotting of proteins for biotinylated (Bio)-dihydrofolate reductase and PBL27 (Bio-DR and Bio-PBL27, respectively) as well as FLAG-tagged CRK2, CRK3, CPK3 and CPK13 (FLAG-CRK2, FLAG-CRK3, FLAG-CPK3 and FLAG-CPK13, respectively) were synthesized using a cell-free system. Total proteins were subjected to 10% SDS-polyacrylamide gel electrophoresis (SDS-PAGE) and immunoblotted with the respective antibodies (α-Biotin and α-FLAG).
